# Protective effects of deferasirox and N-acetyl-L-cysteine on iron overload-injured bone marrow

**DOI:** 10.1590/1414-431X20176087

**Published:** 2017-10-19

**Authors:** J.C. Shen, Y.C. Zhang, M.F. Zhao

**Affiliations:** 1 Department of Hematology, Affiliated Hospital of Logistics University of People's Armed Police Forces, Tianjin, China; 2 Department of Biotherapy, Affiliated Hospital of Logistics University of People's Armed Police Forces, Tianjin, China; 3 Department of Hematology, The First Central Hospital, Tianjin, China

**Keywords:** Iron overload, Bone marrow, Hematopoietic stem/progenitor cells, Deferasirox, N-acetyl-L-cysteine

## Abstract

Using an iron overload mouse model, we explored the protective effect of deferasirox (DFX) and N-acetyl-L-cysteine (NAC) on injured bone marrow hematopoietic stem/progenitor cells (HSPC) induced by iron overload. Mice were intraperitoneally injected with 25 mg iron dextran every 3 days for 4 weeks to establish an iron overload (Fe) model. DFX or NAC were co-administered with iron dextran in two groups of mice (Fe+DFX and Fe+NAC), and the function of HSPCs was then examined. Iron overload markedly decreased the number of murine HSPCs in bone marrow. Subsequent colony-forming cell assays showed that iron overload also decreased the colony forming capacity of HSPCs, the effect of which could be reversed by DFX and NAC. The bone marrow hematopoiesis damage caused by iron overload could be alleviated by DFX and NAC.

## Introduction

Several patients with ineffective hematopoiesis and cytopenia, such as aplastic anemia (1), myelodysplastic syndromes (MDS) (2,3), myelofibrosis (4–6), and β-thalassemia (7,8), require multiple erythrocyte transfusions leading to iron overload (9,10). The clinical characterization of iron overload includes hepatic dysfunction, cardiac dysfunction, glucose intolerance, arthropathy, sexual dysfunction and fatigue (11–17).

Recently, it was reported that rapid accumulation of excessive iron negatively affects the hematopoietic system by damaging hematopoietic cells and hematopoietic microenvironment (18). Some clinical evidence indicates that iron chelation therapy (ICT) can improve hematopoiesis and exert a survival benefit in iron overload patients with low-risk MDS (18–22). Deferasirox (DFX) is an oral iron chelator used to treat iron overload. Several studies indicate that DFX can efficiently improve the hematologic parameters, though the exact mechanism is still unknown (6,22–26). On the other hand, our previous studies indicate that iron overload injures hematopoietic stem cells (HSCs) and mesenchymal stem cells (MSCs) via reactive oxygen species (ROS)-related signaling proteins and subsequent increased cell apoptosis and decreased cell proliferation (18,27–30). Using an antioxidant such as N-acetyl-L-cysteine (NAC) to decrease ROS levels partially attenuates bone marrow mononuclear cell (BMMNC) and umbilical cord-derived MSC injury *in vitro* (14,31).

Despite the above findings, more evidence is needed to determine if iron overload impairs hematopoietic function by enhancing oxidative stress and how DFX and NAC exert their protective effects *in vivo*. Therefore, in this study, we established an iron overload model. Then, we investigated the general characteristics of bone marrow HSPC and the related signaling pathway in this process. Finally, we assayed for the effects of DFX and NAC in this model.

## Material and Methods

### Animal model and treatment

C57BL/6-Ly-5.1 (Ly5.1) male mice were obtained from the Institute of Peking University Health Science Center (Beijing, China). The mice were bred at a certified animal care facility in the Institute of Radiation Medicine of Peking Union Medical College (PUMC). All mice were used at approximately 6–8 weeks of age, and the average weight was 20 g.

First, the iron overload mouse model was established by intraperitoneal injection of varying doses (12.5, 25, or 50 mg) of iron dextran (Sigma-Aldrich, USA) in 1 mL saline every 3 days for 2, 4, or 6 weeks. Ultimately, we chose 25 mg/mL as the final dose (18). Next, the mice were randomly divided into four groups: control group, iron overload (Fe) group (25 mg/mL), Fe+DFX (Novartis, Switzerland) group, and Fe+NAC (Sigma-Aldrich) group. The experimental groups were intraperitoneally injected with 25 mg of iron dextran (Sigma-Aldrich) twice a week for 4 weeks. Control group mice were injected with the same volume of saline (Sigma-Aldrich). The DFX powder (Novartis) was suspended in 0.5% aqueous Klucel hydroxypropylcellulose by ultrasonication, and Fe+DFX group mice received 125 mg/kg DFX by gavage 5 days per week. For NAC (Sigma-Aldrich) treatment, mice were given 40 mM NAC (Sigma-Aldrich) in their drinking water. The water bottles were exchanged twice weekly with a freshly prepared NAC solution (31). All treatments met local regulations and ethical requirements.

### Identification of iron overload mouse model

The deposition of iron into LSK cells (Lin-Sca-1+c-kit+) was assessed using hematoxylin and eosin (HE) staining and Perls' iron staining. Additionally, the labile iron pool (LIP) level of LSKs was measured using a Calcein-AM fluorescent dye (Sigma-Aldrich) (32). The LSKs were analyzed using a flow cytometer with the mean fluorescence intensity (MFI) calculated by the Cell Quest software (BD Bioscience, USA).

### BMMNC counts

The BMMNCs were flushed from the bones as previously described (31,33,34) and were counted using a pocH-100i hematology analyzer (Sysmex, Japan). Mice were sacrificed by cervical dislocation and then liberally rinsed in a beaker with 100 mL of 70% (v/v) ethanol for 3 min. An incision was made through the skin, and the muscles were dissociated. The muscles and tendons were then cleaned from humeri, tibiae, and femurs. Removal of the epiphyses was performed with sterile scissors. The bone marrow was thoroughly flushed with a syringe needle containing 3 mL of alpha modified eagle medium (α-MEM, Gibco, USA) and used for the following experiments.

### Flow cytometric assay

The BMMNCs were stained with PE-conjugated anti-Ter-119 or the biotin-conjugated antibodies Gr-1 and CD11b; the streptavidin APC-CY7 was incubated with DCFH-DA (10 μM) or calcein-AM (0.125 μM) in a humidified atmosphere of 5% CO_2_ in air at 37°C for 15 min. The hematopoietic progenitor cells (HPCs) (Lineage^-^/Sca-1^-^/c-kit^+^, LSK^-^) and HSCs (Lineage^-^/Sca-1^+^/c-kit^+^; LSK^+^) were analyzed as previously described (14), and the levels of intracellular ROS and LIP were analyzed by measuring the MFI of 2′-7′dichlorofluorescein or calcein using a flow cytometer.

### Colony-forming cell (CFC) assay

CFC assays were performed using BMMNCs in MethoCult GF M3434 methylcellulose medium (Stem Cell Technologies, Canada). Colony-forming unit granulocyte-macrophage (CFU-GM), colony-forming unit erythroid (CFU-E), burst-forming unit erythroid (BFU-E), and colony-forming unit mix (CFU-Mix) were counted on days 5, 7, 9, and 12, respectively, using a microscope according to the manufacturer's protocol.

### Analysis of HSCs and HPCs by flow cytometry and establishment of co-culture system

BMMNCs were incubated with biotin-conjugated rat antibodies specific for murine CD5, Mac-1, CD45R/B220, Ter-119, and Gr-1 for 15 min at room temperature. After washing with PBS twice, the cells were stained with APC-Cy7-conjugated streptavidin,PE-Cy7-conjugated anti-Sca1, and Alexa Fluor 700-conjugated anti-c-kit antibodies (eBioscience, USA) and analyzed by flow cytometry. The ratio of HPCs (LSK^-^) and HSCs (LSK^+^) in BMMNCs was calculated.

### Single-cell colony assay

Sorted CD34^-^/LSK^+^ cells were seeded onto the wells of 96-well round-bottom microplates using a BD FACS (USA) Aria III cell sorter at a density of 1 cell/well. The cells were cultured in 200 mL IMDM supplemented with 10% fetal calf serum, 1% bovine serum albumin, 2 mM L-glutamine, 50 mM 2-b-mercaptoethanol, 10 ng/mL stem cell factor, 10 ng/mL thrombopoietin, and 10 ng/mL IL-3, as described previously (14). After 14 days of culture, the colonies of cells with ≥50 cells/well were scored under an inverted microscope. The results are reported as the number of colonies per 20 wells.

### Statistical analysis

All experiments were performed at least three times. The results are reported as means±SD. Multiple group comparisons were performed using analysis of variance (ANOVA). Differences were considered to be statistically significant at α=0.05, and Bonferroni correction was applied for multiple comparison tests. Analyses were performed with the GraphPad Prism program (GraphPad Software, Inc., USA).

### Ethics statement

This study was approved by the PUMC Ethics Committee (No. 2012-0504). Evaluation of the handling of the experimental animals included: 1) the experiment, in which the needs of the animals were fully considered, including physiological (adequate food, water, temperature, and illumination), environmental, psychological, and social needs (socially raised, 4–6 animals per cage, avoiding fatigue and overstimulation). The outcomes of the preliminary experiment and the primary literature were taken into consideration to rationally design the sample size and operation standards. 2) Daily observation was performed to prevent the animals from anger, comfortlessness, fear, nervousness, pain or damage, as well as to maintain them at baseline status. Abuse and excessive or incorrect medication were avoided. For subcutaneous injection, narcotics were not provided. For tail vein injection, intraperitoneal anesthesia was given to alleviate pain. 3) At the end of the experiment, the animals were sacrificed within 15 s to avoid nervousness of the other animals.

## Results

### Establishment of BM iron overload mouse model

**Figure 1.**
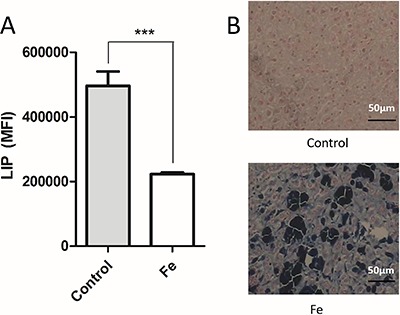
Establishment of an iron overload (Fe) mouse model. *A*, labile iron pool (LIP) level in bone marrow mononuclear cells after 25 mg/mL iron dextran treatment, mean fluorescence intensity (MFI). Data are reported as means±SD. ***P<0.001, ANOVA. *B*, Bone marrow analyzed by Perl's iron staining (×1000).

To confirm the efficacy of bone marrow iron overload mouse model, the LIP levels of the BMMNC were evaluated. When the mice were injected with 25 mg/mL iron dextran for 4 weeks, the calcein AM fluorescence intensity of BMMNC in the iron overload (Fe) group (MFI: 229,774±28,423) was lower than the control group (MFI: 496,300±76,698; P<0.05; Figure 1A), which indicated a higher LIP level in the bone marrow of Fe group mice. Furthermore, bone marrow iron deposits were assessed at the end of fourth week. Iron deposits were easily observed in the bone marrow of Fe group mice (Figure 1B). These results demonstrated that the experimental murine model reflected an iron overload pathogenic condition.

### Ratios of HPCs (LSK^-^) and HSCs (LSK^+^) in iron overload BMMNCs

**Figure 2.**
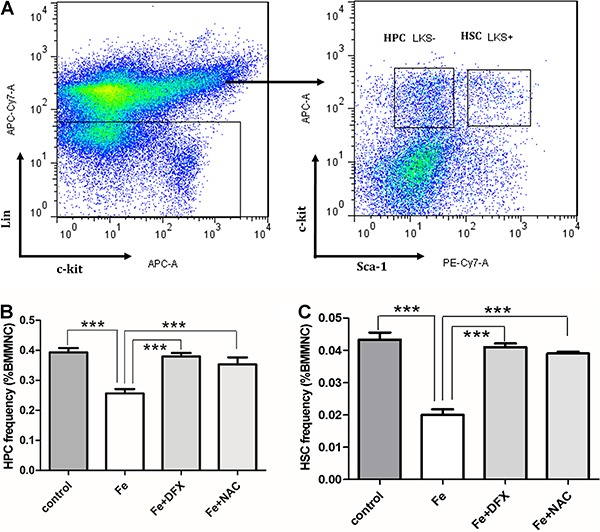
Flow cytometric profile of Lin-Sca-1+c-kit (LKS)^-^ hematopoietic progenitor cells (HPCs) and LKS^+^ hematopoietic stem cells (HSCs) cells (*A*). Decreased ratio of HPCs (*B*) and HSCs (*C*) in iron overload (Fe) bone marrow mononuclear cells (BMMNCs) reversed by deferasirox (DFX) or N-acetyl-L-cysteine (NAC). Data are reported as means±SD. ***P<0.001 (ANOVA).

The ratios of HPCs and HSCs in bone marrow were analyzed by flow cytometry (Figure 2A), and we found that iron overload significantly decreased the frequency of HPCs and HSCs. Notably, this effect was totally reversed by treatment with DFX or NAC (Figure 2B and C, P<0.001 compared to the Fe group). Furthermore, there was no significant difference in the number of HPCs and HSCs between Fe+DFX and Fe+NAC groups, which indicated a similar effect of DFX and NAC.

### LIP levels of LSK^+^cells

**Figure 3.**
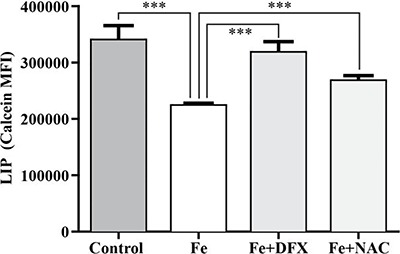
Labile iron pool (LIP) levels of Lin-Sca-1+c-kit (LSK)^+^ in iron overload mice (Fe) significantly increased compared to the control group and were reversed by the administration of deferasirox (DFX) or N-acetyl-L-cysteine (NAC) in mean fluorescence intensity (MFI). Data are reported as means±SD. ***P<0.001, ANOVA.

The LIP levels of Fe mice were significantly increased (MFI: 229,774±28,423; P<0.001) compared to the control group (Figure 3). However, this was significantly reversed by the administration of DFX (MFI: 304,585±3,899) and NAC (MFI: 317,429±19,778) compared to the Fe group (P<0.001).

### ROS levels of LSK^+^cells

**Figure 4.**
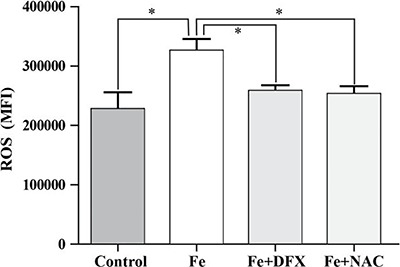
Reactive oxygen species (ROS) levels of Lin-Sca-1+c-kit (LSK)^+^ cells in iron overload (Fe) mice and after the administration of deferasirox (DFX) or N-acetyl-L-cysteine (NAC), in mean fluorescence intensity (MFI). Data are reported as means±SD. *P<0.05 (ANOVA).

The ROS levels of LSK^+^ cells significantly increased in the Fe mice compared to the control group (P<0.05), whereas DFX or NAC treatment significantly inhibited the production of ROS in iron overloaded HSPC compared to the Fe group (P<0.05, Figure 4).

### Bone marrow CFCs

**Figure 5.**
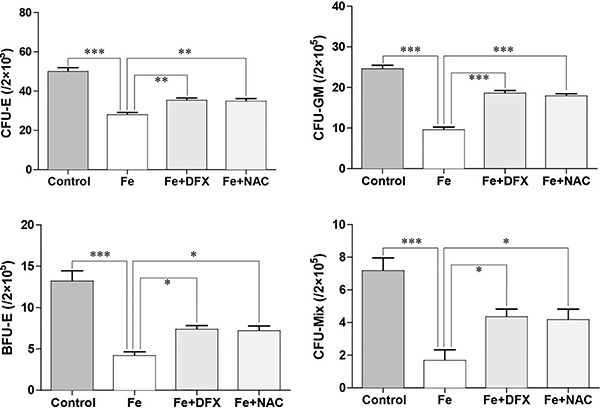
Results of colony-forming unit erythroid (CFU-E), granulocyte-macrophage (CFU-GM), burst-forming unit erythroid (BFU-E), and colony-forming unit mix (CFU-Mix) assays of bone marrow of iron overload (Fe) mice and after the administration of deferasirox (DFX) or N-acetyl-L-cysteine (NAC). Data are reported as means±SD. *P<0.05, **P<0.01, and ***P<0.001 (ANOVA).

**Figure 6.**
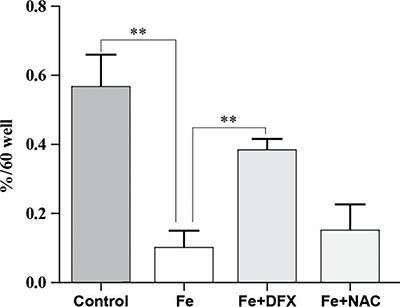
Clonogenic capacity of hematopoietic stem/progenitor cells (HSPCs) in iron overload (Fe) mice and after the administration of deferasirox (DFX) or N-acetyl-L-cysteine (NAC). Data are reported as means±SD. **P<0.01 (ANOVA).

BMMNCs derived from each group were grown in triplicate in M3434 semi-solid media. As shown in Figure 5, CFCs counts (CFU-E, CFU-GM, BFU-E, and CFU-mix) in the Fe group were significantly decreased compared to the control group (all P<0.001), and this effect was significantly reversed by DFX and NAC compared to the Fe group (all P<0.05). Furthermore, the clonogenic capacity of HSPCs was decreased by iron overload (P<0.01), and this effect was improved after administration of DFX (P<0.01). However, no obvious improvement was observed in the NAC group (Figure 6; P>0.05 compared to the control group).

## Discussion

Transfusional iron overload refers to excessive iron deposition in the body, which can lead to abnormal hematopoietic function. In this study, using a novel iron overload mouse model, we observed that excessive iron deposition decreased the number of bone marrow HSPCs. Furthermore, iron overload affected the function of HSPCs, as indicated by a significant reduction of CFUs number and a decreased single-cell clone formation capacity. Importantly, all of these iron overload-induced damages were reversible via iron-chelation (DFX) or anti-oxidation (NAC) treatment.

Clinical evidence shows that ICT can improve hematological parameters and decrease transfusion requirements in MDS patients (35). As an iron chelating agent, deferoxamine (DFO) has been widely used for the treatment of secondary iron overload and displays an anti-proliferative activity against a wide range of tumors (36,37). However, its high hydrophilicity and short half-life limit the effect of DFO. Serious *Listeria monocytogenes* infections have also been reported in secondary iron overload mice intraperitoneally injected with DFO (38). Overcoming these limitations, the oral agent DFX also shows significant anti-proliferative activity against hepatoma and myeloid malignant tumors (39). In addition, it was found that iron overload significantly delays hematopoietic recovery after bone marrow transplantation, which indicates that iron overload may impact the hematopoietic microenvironment of bone marrow (23). We recently confirmed the toxic effect of free iron on HSPCs and supported the protective efficiency of DFX in hematopoietic recovery (14,25).

The inhibitory effect of iron overload is mainly related to the activation of intracellular ROS. It has been confirmed that iron overload occurring through mediated oxidative stress can result in tissue damage in a diabetic animal model (38). ROS is mainly created through NADPH in the mitochondria (40). Under normal physiological conditions, intracellular ROS stays at a low level, but under stress or damage conditions, increasing levels of active oxygen lead to protein, cell membrane, and DNA damage, which can result in aging and apoptosis of hematopoietic cells (18). We found that iron overload increases the ROS level of HSPCs and impacts hematopoietic function, whereas the anti-oxidant NAC improves this situation. These data confirm the ROS involvement in iron overload-induced HSPC damage and indicate that NAC is an option for iron overload therapy.

In conclusion, the iron overload mouse model was successfully established for further experiments. Iron overload-induced damage to bone marrow HSPCs could be partially improved by the iron-chelating agent DFX or the antioxidant NAC.
